# Antibody-Dependent Enhancement of Porcine Reproductive and Respiratory Syndrome Virus Infection Antagonizes the Secretion of Type I Interferons in Porcine Alveolar Macrophages by Interfering with the Retinoic Acid-Inducible Gene I/Melanoma Differentiation-Associated Gene 5 Pathway via Fc Gamma Receptor I

**DOI:** 10.3390/v17091277

**Published:** 2025-09-20

**Authors:** Liujun Zhang, Aiyang Wang, Weizhen Chen, Xing Feng, Bo Wang, Shaojun He, Hongjie Fan

**Affiliations:** 1 College of Animal Science, Anhui Science and Technology University, Chuzhou 233100, China; 2 Anhui Province Key Laboratory of Animal Infectious Disease Prevention and Control, Chuzhou 233100, China; 3 Anhui Province Key Laboratory of Animal Nutrition Regulation and Health, Chuzhou 233100, China; 4 Anhui Engineering Technology Research Center of Pork Quality Control and Enhancement, Chuzhou 233100, China

**Keywords:** PRRSV, ADE, FcγRI, RIG-I, MDA5, Type I IFNs

## Abstract

Type I interferons (IFNs), mainly IFN-α and IFN-β, play an essential role in defending against viral invasion by inducing the host’s innate antiviral response. Porcine reproductive and respiratory syndrome virus (PRRSV) is known to impair the IFN responses of infected hosts through the antibody-dependent enhancement (ADE) infection pathway, but the precise mechanisms employed are poorly understood. In this study, we showed that PRRSV alone induced a strong secretion of IFN-α and IFN-β in infected porcine alveolar macrophages (PAMs) by activating the retinoic acid-inducible gene I (RIG-I)/melanoma differentiation-associated gene 5 (MDA5) signaling pathway. By contrast, ADE infection of PRRSV significantly down-regulated the production levels of IFN-α and IFN-β in PAMs by negatively regulating the RIG-I/MDA5 signaling pathway and considerably enhancing the replication level of PRRSV in PAMs. Next, small interfering RNA (siRNA) experiments revealed that Fc gamma receptor I (FcγRI) was responsible for the ADE infection of PRRSV in PAMs. In addition, we observed that FcγRI mediated the potent inhibition of IFN-α and IFN-β production through blocking the activation of the RIG-I/MDA5 signaling pathway in PAMs. Further, we found that FcγRI effectively inhibited PRRSV-induced synthesis of IFN-α and IFN-β by negatively regulating PRRSV-induced activation of the RIG-I/MDA5 signaling pathway in PAMs and significantly increased the viral production of PRRSV in PAMs. In conclusion, these results suggest that ADE infection of PRRSV may antagonize the secretion of type I IFNs (IFN-α/β) by interfering with the RIG-I/MDA5 pathway via FcγRI in PAMs, thereby facilitating the proliferation level of PRRSV in PAMs.

## 1. Introduction

The interferons (IFNs) are a class of pleiotropic cytokines with powerful biological functions such as antiviral, anti-tumor, growth inhibition, and immune regulation. Type I IFNs (IFN-α/β) are typical antiviral cytokines that can interfere with viral replication within the host [1]. Type I IFNs are usually induced when virus infection is detected by pattern recognition receptors (PRRs), including retinoic acid-inducible gene I (RIG-I)-like receptors (RLRs) or toll-like receptors (TLRs) [2]. RIG-I and melanoma differentiation-associated gene 5 (MDA5) are two key RLRs for recognizing double-stranded (ds) RNA. Upon recognition, RIG-I/MDA5 can recruit the mitochondrial antiviral signaling protein (MAVS), which leads to downstream activation of tumor necrosis factor receptor-associated factor (TRAF)-associated nuclear factor-κB (NF-κB) activator-binding kinase 1 (TBK1), with the subsequent activation of IFN regulatory factor 3 (IRF3). Finally, activated IRF3 induces the production of type I IFNs [3,4].

Immunoglobulin G (IgG) Fc receptors (FcγRs) are a family of receptors universally expressed on most immune leukocyte subsets. Based on their capacity to bind the Fc fragments of IgGs in different conformations, the FcγR system is divided into two distinct types (type I or type II FcγRs) [5]. Type I FcγRs belong to the Ig superfamily and consist of several activating receptors (FcγRI, FcγRIIa/c, FcγRIIIa/b, and FcγRIV) and one inhibitory (FcγRIIb) receptor. Type II FcγRs are members of the C-type lectin receptor family, such as CD23 and CD209 [6,7]. From the perspective of structural characteristics, activating FcγRs are characterized by the presence of an intracellular immunoreceptor tyrosine-based activation motif (ITAM) in the receptor α-chain (FcγRIIa/c) or at the associated FcR γ-chain (FcγRI and FcγRIIIa). By contrast, the sole inhibitory FcγRIIb has an immunoreceptor tyrosine-based inhibitory motif (ITIM) in its cytosolic tail [8]. Crosslinking of activating or inhibitory FcγRs by ligands activates immunostimulatory or immunosuppressive signals, respectively, thereby determining the outcome of IgG-mediated immunotherapy, inflammation, and immunity [9]. FcγR engagement can trigger the activation of downstream immunomodulatory pathways to mediate multiple effector functions, including cytotoxicity and phagocytosis of IgG-coated immune complexes (ICs), synthesis and release of inflammatory factors and chemokines, activation and differentiation of T/B cells and antigen-presenting cells, survival of plasma cells, and modulation of antibody responses, etc. [10,11]. These functions highlight the potential of FcγRs in regulating adaptive immune responses. In addition, FcγRs can participate in innate antiviral immunity by influencing the IFN response programs in different myeloid cell types [12,13,14,15]. However, the precise mechanisms by which activating/inhibitory FcγRs regulate the expression of type I IFNs remain to be better clarified.

The antibody response with neutralizing activity induced by infection or vaccination can effectively prevent the occurrence of infectious diseases. Nevertheless, there are also examples where sub-neutralizing or non-neutralizing antibodies enhance viral entry into host cells, thereby increasing the severity of infection and disease. This phenomenon is termed antibody-dependent enhancement (ADE) [16,17]. Many viruses exhibit the ADE effects, such as Dengue virus (DENV), Ross River virus (RRV), influenza virus, Ebola virus, Zika virus, Middle East respiratory syndrome (MERS) virus, severe acute respiratory syndrome (SARS) virus, human immunodeficiency virus (HIV), and porcine reproductive and respiratory syndrome virus (PRRSV) [18,19,20]. Early studies have shown that ADE events occur in FcγR-bearing phagocytic cells. It is hypothesized that preexisting anti-virus IgG antibodies form antibody-virion ICs that bind to FcγRs [21]. Subsequently, FcγRs not only promote the internalization of viral particles into cells through the phagocytosis pathway but also facilitate immune evasion within cells through specific mechanisms, ultimately leading to increased virus replication [22,23,24].

PRRSV is an enveloped positive-stranded RNA virus belonging to the *Arteriviridae* family and preferentially replicates in porcine alveolar macrophages (PAMs) [25]. This virus can infect PAMs through typical cell receptors and significantly enhance its infectivity for cells expressing FcγRs by utilizing the ADE mechanism [26,27,28]. Over the past nearly 40 years, PRRSV infection has brought heavy disasters to the global pig farming industry, yet effective prevention and control measures remain elusive [29]. The current main prevention strategies include the use of inactivated vaccines and modified live attenuated vaccines. However, they cannot provide adequate protection against the challenges of heterologous strains [30,31]. More unfortunately, ADE not only exacerbates the severity of PRRSV infection but also becomes a significant obstacle in the development of effective PRRSV vaccines [32,33,34]. Furthermore, some early reports have demonstrated that FcγR-mediated ADE of PRRSV suppresses the host’s innate antiviral immunity by down-regulating the production of type I IFNs. Although the mechanism by which ADE inhibits the levels of type I IFNs is incompletely understood, these studies suggested that the impairment of IFN response by ADE results in innate immunosuppression and subsequent persistent infection that are characteristics of PRRSV [28,35,36,37]. A clear understanding of the events of virus infection through the FcγR pathway is crucial for formulating effective intervention measures against PRRSV infection. Therefore, the objective of this study was to explore the mechanisms of innate immune suppression during FcγRI-mediated ADE of PRRSV infection in PAMs.

## 2. Materials and Methods

### 2.1. Cell Culture and Virus Strain

PAMs isolated from the postmortem bronchoalveolar lavage fluid of six-week-old PRRSV-negative pigs were grown in RPMI-1640 medium (Sangon Biotech, Shanghai, China) containing 10% (vol/vol) fetal bovine serum (FBS) (Procell System, Wuhan, China) at 37 °C with 5% CO_2_. The North American-type (type 2) PRRSV strain AF-9 (GenBank accession number: PX068332) used in this study was propagated in Marc-145 cells. The median tissue culture infective dose (TCID_50_)/mL of PRRSV AF-9 was titrated on Marc-145 cells using the Reed-Muench method [38].

### 2.2. Antibodies and Reagents

Pig anti-PRRSV AF-9 strain-specific polyclonal antibody (pAb) [Enzyme-linked immunosorbent assay (ELISA) antibody titer: 1:7200] was obtained from the hyper-immune serum of pigs inoculated with inactivated and purified AF-9 strain virions. Rabbit anti-pig FcγRI specific pAb (ELISA antibody titer: 1:12800) was generated by immunizing rabbits with the extracellular domain protein of pig FcγRI. The IgGs were purified from each pAb by diethylaminoethanol chromatography and preserved in our laboratory. Pig-negative IgG (PNI) and rabbit-negative IgG (RNI) were extracted from the sera of PRRSV-negative piglets and the sera of clean-level white rabbits, respectively. Rabbit monoclonal antibodies (mAbs) against RIG-I, MDA5, phospho-TBK1 at Ser^172^, and phospho-IRF3 at Ser^396^ were purchased from CST (Boston, MA, USA). Rabbit pAs specific for TBK1, IRF3, and β-actin were provided by Absin (Shanghai, China). PRRSV nucleocapsid (N) protein rabbit pAb, MAVS rabbit pAb, TRAF3 rabbit pAb, and horseradish peroxidase (HRP)-conjugated anti-rabbit IgG antibody were from Bioss (Beijing, China), Invitrogen (Carlsbad, CA, USA), LifeSpan (Salt Lake City, UT, USA), and EarthOx (San Francisco, CA, USA), respectively. Polyinosinic-polycytidylic acid [i.e., Poly (I: C)], a synthetic analog of dsRNA, was the product of InvivoGen (Toulouse, France). Lipofectamine 3000 transfection reagent was obtained from Invitrogen.

### 2.3. ADE Assay of PRRSV Infection in PAMs

Enhanced PRRSV AF-9 strain/pig anti-PRRSV AF-9 IgG antibody infectious immune complexes (PRRSV-ICs) and un-enhanced PRRSV AF-9 strain/pig-negative IgG (PNI) admixtures (PRRSV-PNI) were prepared as previously described by Zhang et al. [28]. Briefly, 2000 TCID_50_/mL of PRRSV AF-9 suspensions were thoroughly mixed and incubated with 765 μg/mL of pig anti-PRRSV AF-9 IgG or PNI in an equal volume for 1 h at 37 °C to generate PRRSV-ICs or PRRSV-PNI, respectively. ADE infection of PRRSV AF-9 strain into PAMs was conducted as follows: PAM monolayers cultured in 24-well plates (Corning, NY, USA) were incubated with PRRSV at a titer of 200 TCID_50_, PRRSV-ICs, or PRRSV-PNI. After incubation for 1 h at 37 °C, the cells were washed and then cultivated in a fresh complete growth medium. The infected cells and supernatants collected at 12 h and 24 h post-infection were used in subsequent experiments, including relative quantitative RT-PCR (qRT-PCR), ELISA, Western blot, and detection of viral RNA copies and tites. In this experiment, the control groups were set, PAMs stimulated with poly (I: C) at a 100 μg/mL concentration, and the mock-infected PAMs.

### 2.4. Silencing Assay of the FcγRI Gene in PAMs

Small interfering RNA (siRNA) against porcine FcγRI (Sense: GCCUUGAGGUGU-CAUGGAUTT/Anti-sense: AUCCAUGACACCUCAAGGCTT) and negative control siRNA (Sense: UUCUCCGAACGUGUCACGUTT/Anti-sense: ACGUGACACGUUCGG-AGAATT) were synthesized by GenePharma (Shanghai, China). PAM monolayers seeded in 6-well plates (Corning) were transfected with 50 pmol of FcγRI siRNA or negative control siRNA using Lipofectamine 3000 transfection reagent. The cells were harvested after 24, 36, 48, and 60 h of transfection, and relative qRT-PCR and Western blot analyses were used to assess the silencing effect of FcγRI siRNA. Next, at 36 h post-transfection, the cells were infected with PRRSV-ICs. The cells and supernatants were collected at 12 h and 24 h post-infection to determine viral RNA copies and titers.

### 2.5. Activation Assay of FcγRI in Healthy PAMs

PAM monolayers prepared in 24-well plates were incubated for 1 h at 37 °C with either anti-FcγRI IgG or rabbit-negative IgG (RNI) at a concentration of 200 μg/mL of each antibody. The control groups were PAMs stimulated with poly (I: C) (100 μg/mL) and the untreated mock PAMs. Then, the cells were washed and further grown in a fresh, complete-growth medium. The cells and supernatants harvested at 12 h and 24 h post-treatment were subjected to relative qRT-PCR, ELISA, and Western blot.

### 2.6. Activation Assay of FcγRI in Poly (I: C)-Stimulated PAMs

PAM monolayers kept in 24-well plates were pre-incubated for 1 h at 37 °C with either anti-FcγRI IgG (200 μg/mL) or RNI (200 μg/mL). After incubation, the cells were stimulated for 12 h and 24 h with 100 μg/mL of poly (I: C). Poly (I: C)-stimulated PAMs, and the unstimulated mock PAMs acted as the control groups. The cells and supernatants were harvested for relative qRT-PCR, ELISA, or Western blot.

### 2.7. Activation Assay of FcγRI in PRRSV-Infected PAMs

PAM monolayers maintained in 24-well plates were pre-treated for 1 h at 37 °C with either anti-FcγRI IgG (200 μg/mL) or RNI (200 μg/mL). After treatment, the cells were infected for 12 h and 24 h with 200 TCID_50_ of PRRSV. PRRSV-infected PAMs and the uninfected mock PAMs served as the control groups. The cells and supernatants were collected for relative qRT-PCR, ELISA, Western blot, real-time RT-PCR, or virus titration.

### 2.8. RNA Extraction, Real-Time RT-PCR, and Relative qRT-PCR

Total RNAs were isolated from PAMs using TRIZOL (TaKaRa, Beijing, China). The RNAs were converted into cDNA using reverse transcription kits (TaKaRa). Viral RNA copy numbers were monitored by targeting the ORF7 gene of PRRSV using real-time RT-PCR, as previously reported by Zhang et al. [39]. Relative qRT-PCR was performed in the ABI Q5 Real-Time System (Foster City, CA, USA) to measure the mRNA expression levels of immune cytokines, key molecules of the RIG-I/MDA5 signaling pathway, and FcγRI. Amplification reaction parameters were 2 min at 95 °C, followed by 40 cycles of 5 s at 95 °C and 20 s at 60 °C. Transcriptional expression of the target genes was analyzed using the 2^−∆∆CT^ method. The final relative transcript levels of target genes were normalized using β-actin. Results were shown as the fold change in treatment relative to the control. The specific primer sets for IFN-α, IFN-β, RIG-I, MDA5, MAVS, TRAF3, TBK1, IRF3, FcγRI, and β-actin were listed in Table 1.

### 2.9. ELISA Detection of IFN-α and IFN-β Production

Immune cytokine IFN-α and IFN-β levels in collected PAM culture supernatants were quantified using commercial ELISA kits for pig IFN-α (Sigma, St. Louis, MO, USA) and swine IFN-β (Invitrogen), according to the manufacturer’s protocol.

### 2.10. Western Blot Analysis

PAM cell lysates were subjected to 10% sodium dodecyl sulfate-polyacrylamide gel electrophoresis (SDS-PAGE) and then transferred to polyvinylidene difluoride (PVDF) membranes (Millipore, Billerica, MA, USA). Following the manufacturer’s instructions, the membranes were blocked in a QuickBlock™ blocking buffer (Beyotime, Shanghai, China). The blocked PVDF membranes were incubated with the appropriate primary antibodies at a suitable dilution as recommended [anti-RIG-I, -MDA5, -TRAF3, -TBK1, -IRF3, -phospho-TBK1 (Ser^172^), and -phospho-IRF3 (Ser^396^) at 1:1000; anti-PRRSV N protein at 1:2000; anti-β-actin at 1:3000; anti-MAVS at 2 μg/mL; anti-FcγRI at 5 μg/mL] at 4 °C overnight and the HRP-labeled anti-rabbit IgG secondary antibody at a dilution of 1:10,000 at room temperature for 1 h. Detection of immunolabeled target proteins was visualized by treating the membranes using the ECL chemiluminescence reagent of GE Healthcare (Boston, MA, USA). The β-action was used as an internal reference to normalize the target proteins.

### 2.11. Statistical Analysis

Statistical comparisons of the present study were conducted by two-way analysis of variance (ANOVA) using GraphPad Prism 5.0 software package (San Diego, CA, USA). Results were presented as means ± standard deviations (SD) of data from at least three independent experiments, with a *p*-value less than 0.05 considered statistically significant.

## 3. Results

### 3.1. ADE Infection Phenomenon of PRRSV Is Observed in Porcine IgG Antibodies Against PRRSV in PAMs

**Figure 1:**
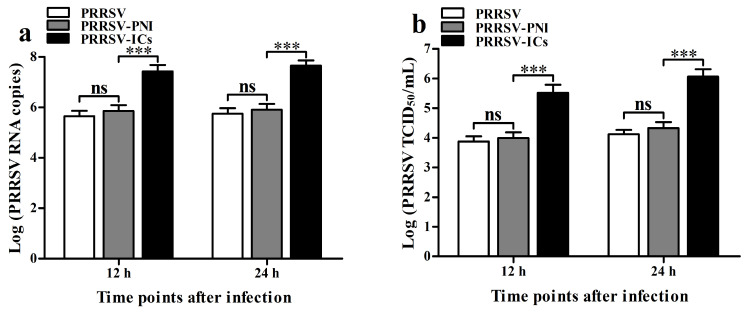

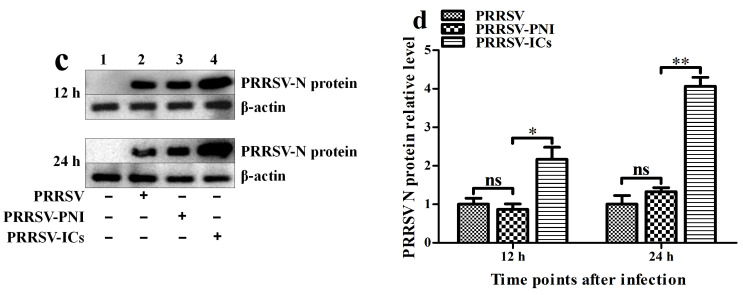
The ADE infection activity of PRRSV in PAMs is mediated by pig anti-PRRSV IgG-specific antibody. PAM cells were infected with PRRSV, PRRSV-ICs, or PRRSV-PNI. Real-time RT-PCR, virus titration, and Western blot determined PRRSV yields. (**a**) Results of real-time RT-PCR detection of PRRSV RNA copy numbers. (**b**) Results of virus titration of PRRSV TCID_50_ infection titers. (**c**) Results of Western blot analysis of PRRSV N protein levels. (**d**) Results of greyscale value analysis of Figure 1c. Data shown are means ± SD from three independent experiments. *** *p* < 0.001, ** *p* < 0.01, * *p* < 0.05, “ns” indicating no significant difference.

To study the effect of pig anti-PRRSV IgG antibody on the replication of PRRSV, we inoculated PRRSV AF-9 strain, PRRSV-ICs, or PRRSV-PNI into PAMs. The viral yields of PRRSV at 12 h and 24 h post-infection were detected using real-time RT-PCR, virus titration, and Western blot. Figure 1 showed that the PRRSV-ICs-infected cell group significantly increased PRRSV RNA copy numbers (Figure 1a) and PRRSV TCID_50_ infection titers (Figure 1b) at 12 h and 24 h post-infection compared to those in the PRRSV-PNI-infected cell group. There were no significant differences in the levels of PRRSV production between the PRRSV-infected cell group and the PRRSV-PNI-infected cell group at 12 h and 24 h after infection. Immunoblot analysis of PRRSV N protein levels in PAMs further supported the above results (Figure 1c,d). These data suggested that the ADE infection activity of PRRSV was present in pig IgG antibodies against PRRSV in PAMs.

### 3.2. PRRSV Induces the Production of Type I IFNs by Positively Regulating the RIG-I/MDA5 Signaling Pathway in PAMs

**Figure 2:**
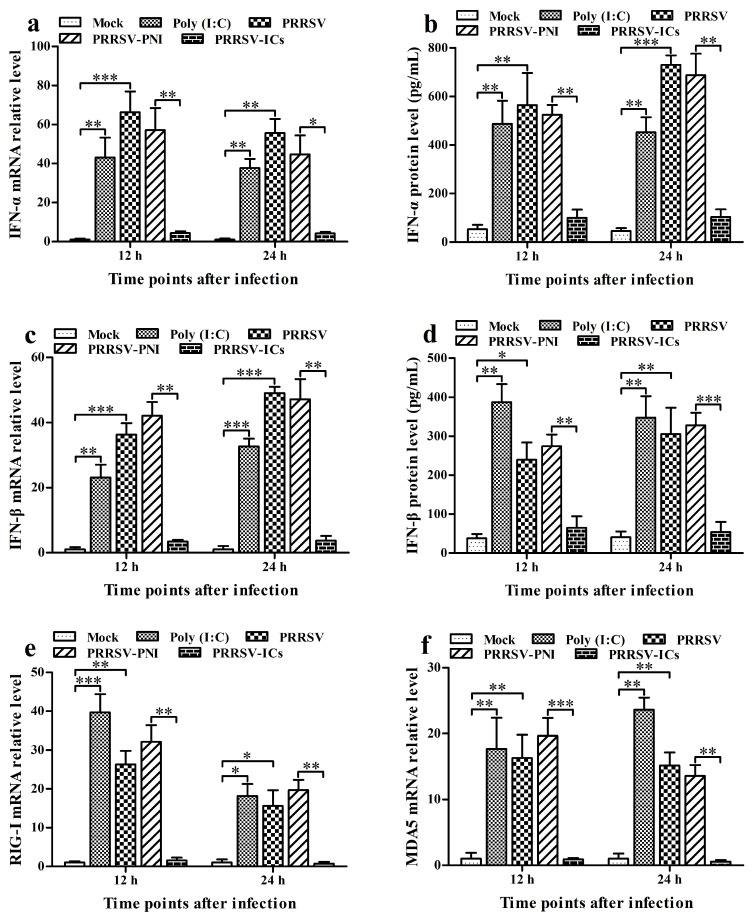

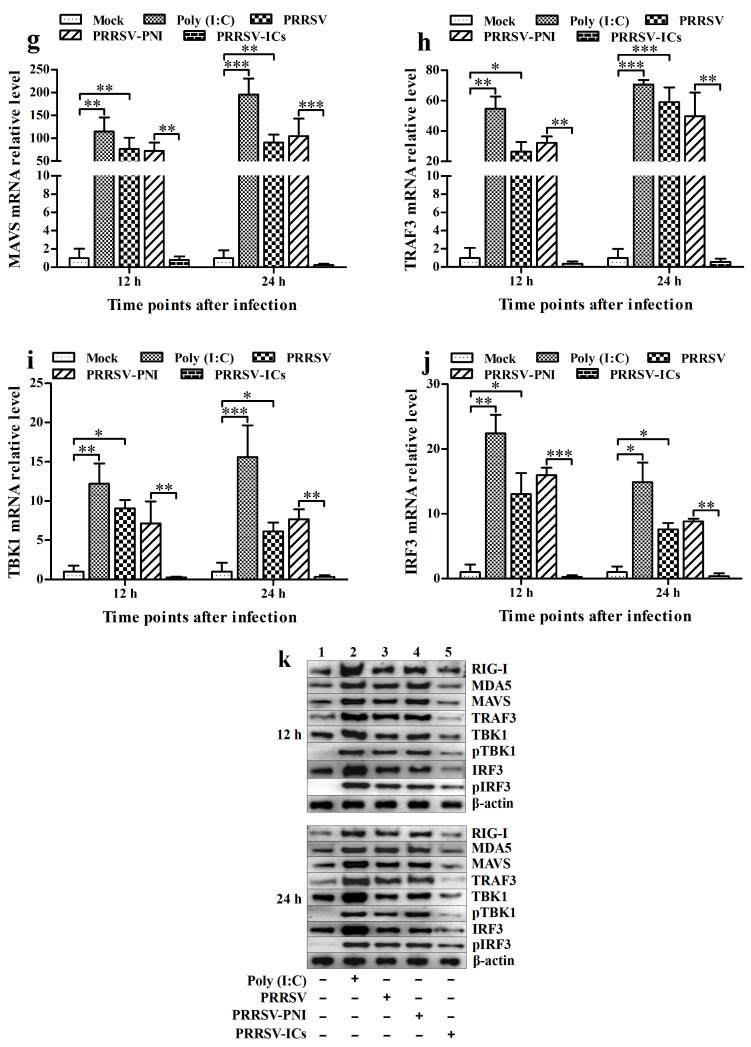

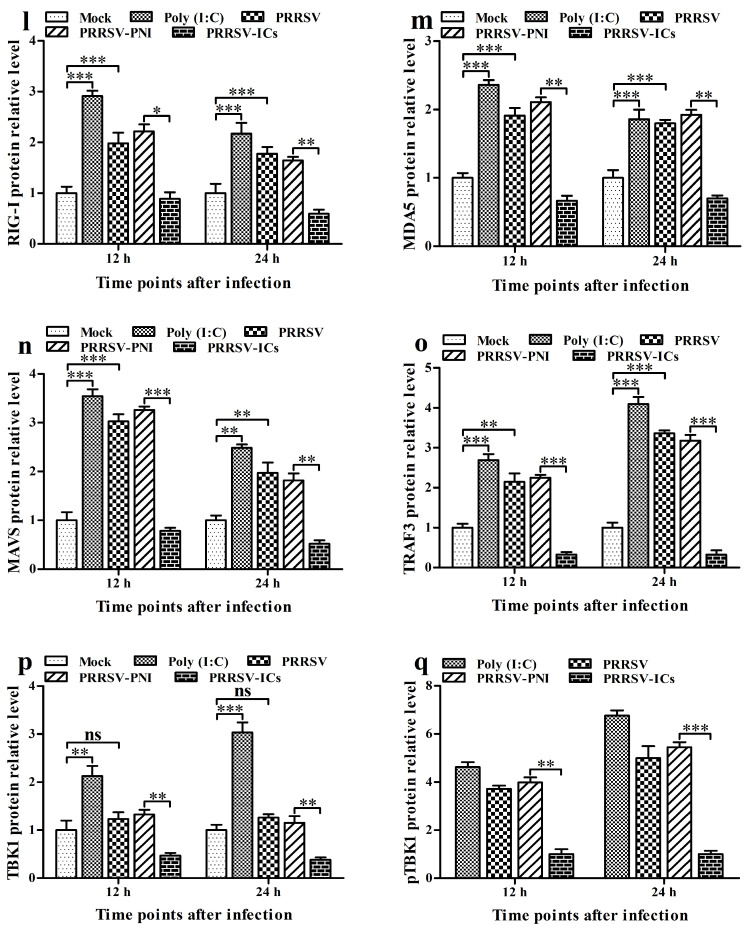

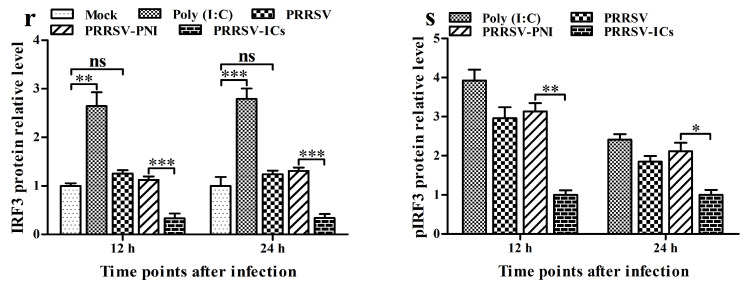
Effect of PRRSV or ADE infection on type I IFN production and the RIG-I/MDA5 signaling pathway in PAMs. PAM cells were infected with PRRSV, PRRSV-ICs, or PRRSV-PNI. Type I IFN expression levels were detected using relative qRT-PCR and ELISA. Expression and phosphorylation levels of key molecules in the RIG-I/MDA5 signaling pathway were measured by relative qRT-PCR and Western blot. (**a**–**d**) Results of relative qRT-PCR or ELISA analysis of IFN-α and IFN-β production. (**e**–**j**) Results of relative qRT-PCR analysis of RIG-I, MDA5, MAVS, TRAF3, TBK1, and IRF3 mRNA levels. (**k**) Results of Western blot analysis of RIG-I, MDA5, MAVS, TRAF3, TBK1, pTBK1, IRF3, and pIRF3 protein levels. (**l**–**s**) Results of greyscale value analysis of Figure 2k. Data shown are means ± SD from three independent experiments. *** *p* < 0.001, ** *p* < 0.01, * *p* < 0.05, “ns” indicating no significant difference.

To investigate the effect of PRRSV on the RIG-I/MDA5 signaling pathway and the expression of type I IFNs, we infected PAMs with PRRSV for the indicated times. Then, we analyzed the activation status of RIG-I/MDA5 pathway key molecules (RIG-I, MDA5, MAVS, TRAF3, TBK1, and IRF3) and the levels of IFN-α and IFN-β. PAMs stimulated by poly (I: C) (an activator of the RIG-I/MDA5 pathway) were set as the positive control group. As shown in Figure 2, the transcript and protein levels of IFN-α, IFN-β, RIG-I, MDA5, MAVS, and TRAF3 in the PRRSV-infected cell group and poly (I: C)-stimulated cell group were significantly elevated at 12 h and 24 h post-infection compared to those in the mock-infected cell group (Figure 2a–h,k–o). Compared with the mock-infected cell group, although TBK1 and IRF3 mRNAs in the PRRSV-infected cell group were significantly up-regulated at 12 h and 24 h post-infection, their proteins did not show a significant change (Figure 2i–k,p,r). In addition, phosphorylation of TBK1 (pTBK1) and IRF3 (pIRF3) was observed in both the PRRSV-infected cell group and poly (I: C)-stimulated cell group at 12 h and 24 h post-infection. At the same time, no pTBK and pIRF3 were detected in the mock-infected cell group at 12 h and 24 h post-infection (Figure 2k,q,s). These data suggested that PRRSV might induce the production of type I IFNs by positively regulating the RIG-I/MDA5 signaling pathway in PAMs.

### 3.3. ADE Infection of PRRSV Down-Regulates the Production of Type I IFNs by Negatively Regulating the RIG-I/MDA5 Signaling Pathway in PAMs

To investigate the impact of PRRSV-ADE infection on the RIG-I/MDA5 signaling pathway and the expression of type I IFNs, we infected PAMs with PRRSV-ICs for the indicated times. Then, we analyzed the activation status of key molecules in the RIG-I/MDA5 pathway and the levels of IFN-α and IFN-β. As illustrated in Figure 2, the transcript and protein levels of IFN-α, IFN-β, RIG-I, MDA5, MAVS, TRAF3, TBK1, and IRF3 in the PRRSV-ICs-infected cell group were significantly diminished at 12 h and 24 h post-infection compared to those in the PRRSV-PNI-infected cell group (Figure 2a–p,r). Meanwhile, the levels of pTBK and pIRF3 in the PRRSV-ICs-infected cell group were significantly lower than those in the PRRSV-PNI-infected cell group at 12 h and 24 h after infection (Figure 2k,q,s). However, there were no significant differences in the expression of IFN-α, IFN-β, RIG-I, MDA5, MAVS, TRAF3, TBK1, and IRF3, as well as the amounts of pTBK and pIRF3, between the PRRSV-infected cell group and the PRRSV-PNI-infected cell group. These data suggest that ADE infection of PRRSV may downregulate the production of type I IFNs by negatively regulating the RIG-I/MDA5 signaling pathway in PAMs.

### 3.4. FcγRI Is Involved in ADE Infection of PRRSV in PAMs

**Figure 3:**
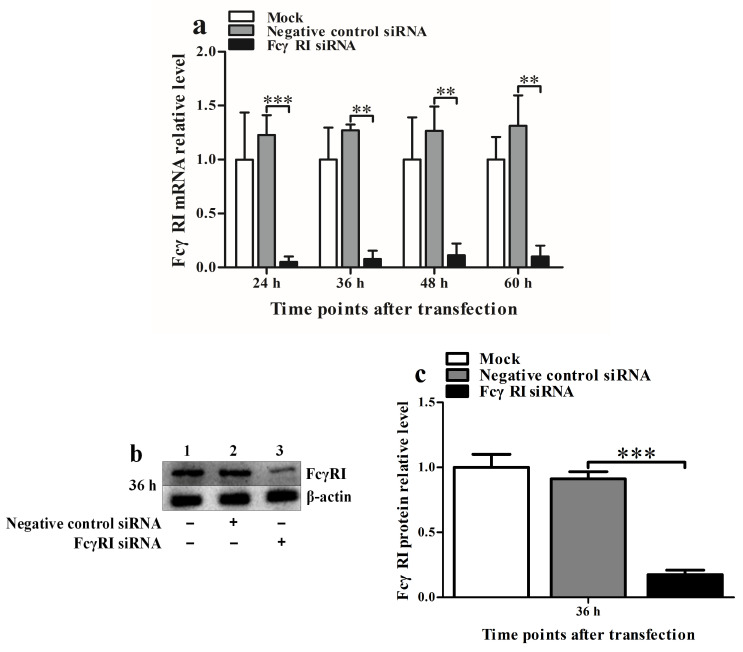
Silencing of FcγRI in PAMs. PAM cells were transfected with FcγRI siRNA or negative control siRNA. FcγRI expression level was detected using relative qRT-PCR and Western blot. (**a**) Results of relative qRT-PCR analysis of FcγRI mRNA level. (**b**) Results of Western blot analysis of FcγRI protein level. (**c**) Results of the greyscale value analysis of Figure 3b. Data shown are means ± SD from three independent experiments. *** *p* < 0.001, ** *p* < 0.01.

**Figure 4:**
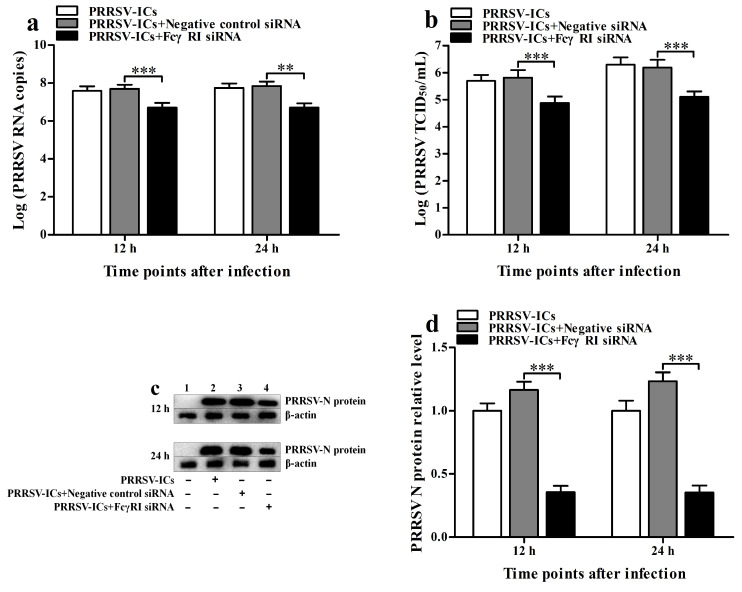
FcγRI is involved in ADE infection of PRRSV in PAMs. PAM cells were transfected with FcγRI siRNA or negative control siRNA for 36 h and then infected with PRRSV-ICs. Real-time RT-PCR, virus titration, and Western blot were used to determine PRRSV yields. (**a**) Results of real-time RT-PCR detection of PRRSV RNA copy numbers. (**b**) Results of virus titration of PRRSV TCID_50_ infection titers. (**c**) Results of Western blot analysis of PRRSV N protein levels. (**d**) Results of greyscale value analysis of Figure 4c. Data shown are means ± SD from three independent experiments. *** *p* < 0.001, ** *p* < 0.01.

To investigate the role of FcγRI in ADE infection of PRRSV, we used siRNA to knock down FcγRI in PAMs and then infected the knocked down cells with PRRSV-ICs at 36 h after knockdown. At 12 h and 24 h after infection, the production of PRRSV in infected cells was determined. As shown in Figure 3, the FcγRI siRNA-transfected cell group significantly downregulated FcγRI mRNA level at 24–60 h after transfection (Figure 3a) and significantly downregulated FcγRI protein level at 36 h after transfection (Figure 3b,c) compared to the negative control siRNA-transfected cell group. Compared with the mock-transfection cell group, the negative control siRNA-transfected cell group did not affect the transcription and translation of FcγRI within the indicated times after transfection. As illustrated in Figure 4, PRRSV-ICs infected cell group transfected with FcγRI siRNA significantly cut down PRRSV RNA copy numbers (Figure 4a), PRRSV TCID_50_ infection titers (Figure 4b), and PRRSV N protein levels (Figure 4c,d) at 12 h and 24 h after infection compared to PRRSV-ICs infected cell group transfected with negative control siRNA. However, the RNA copy numbers, TCID_50_ infection titers, and N protein levels of PRRSV in PRRSV-ICs-infected cells transfected with negative control siRNA were similar to those in PRRSV-ICs-infected cells without siRNA transfection. These data suggested that FcγRI was involved in ADE infection of PRRSV in PAMs.

### 3.5. FcγRI Down-Regulates the Production of Type I IFNs by Negatively Regulating the RIG-I/MDA5 Signaling Pathway in PAMs

**Figure 5:**
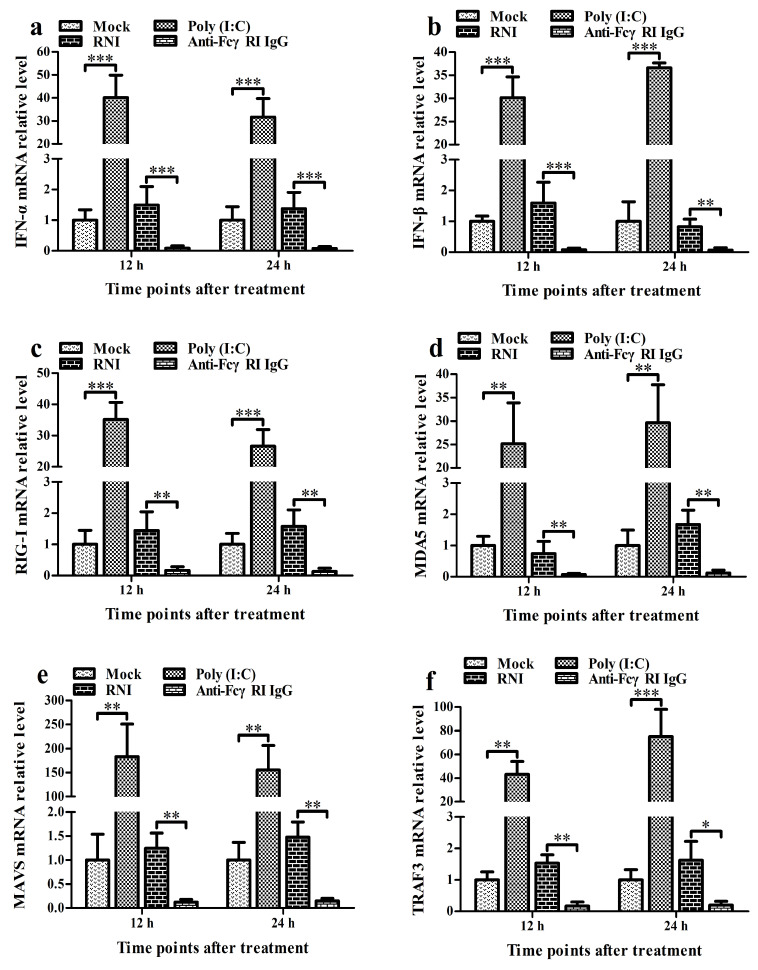

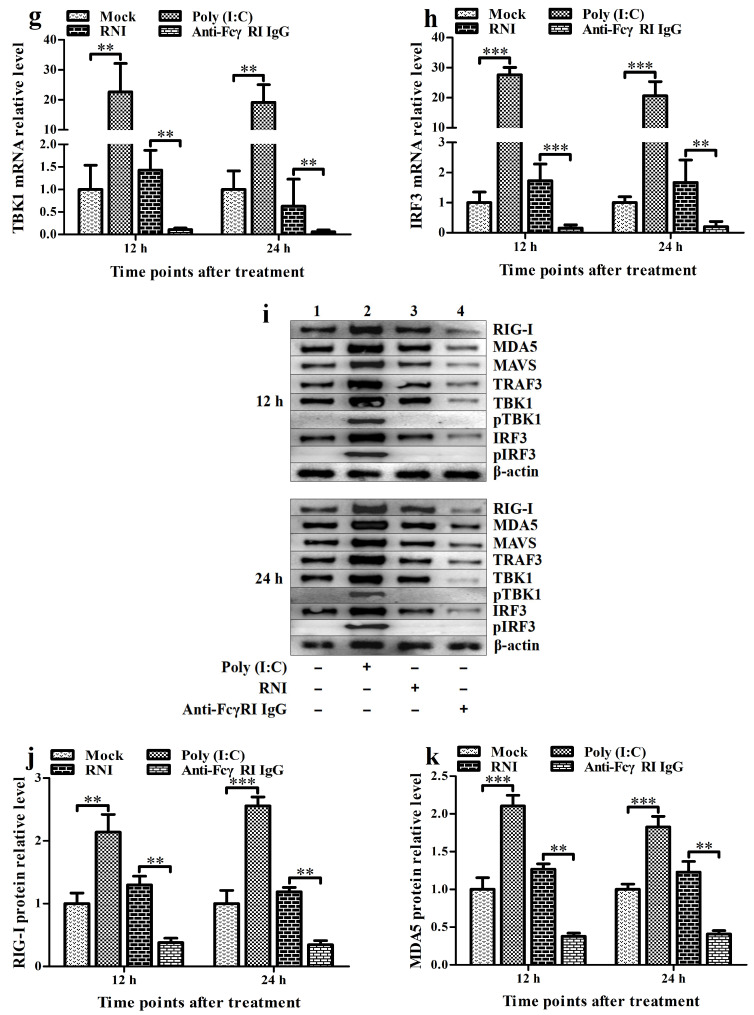

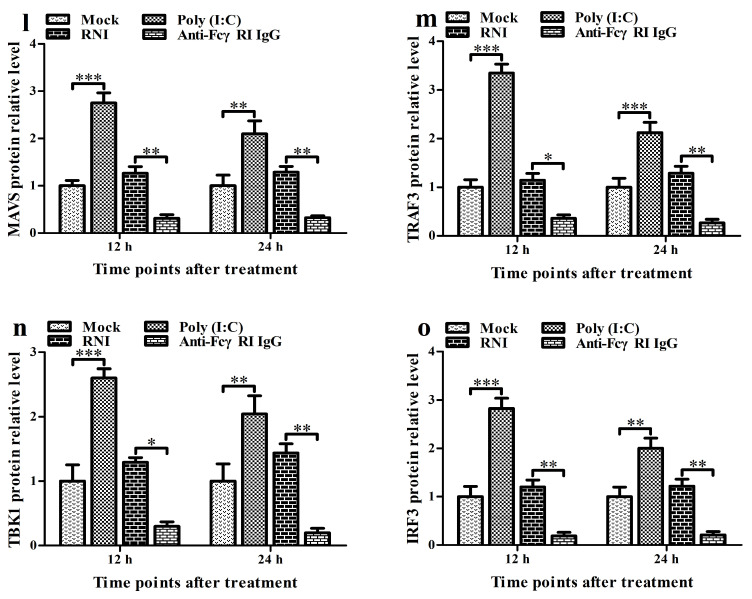
Effect of FcγRI on type I IFN production and the RIG-I/MDA5 signaling pathway in PAMs. PAM cells were treated with anti-FcγRI IgG or RNI. Type I IFN expression levels were detected using relative qRT-PCR and ELISA. Expression and phosphorylation levels of key molecules in the RIG-I/MDA5 signaling pathway were measured by relative qRT-PCR and Western blot. (**a**–**h**) Results of relative qRT-PCR analysis of IFN-α, IFN-β, RIG-I, MDA5, MAVS, TRAF3, TBK1, and IRF3 mRNA levels. (**i**) Results of Western blot analysis of RIG-I, MDA5, MAVS, TRAF3, TBK1, pTBK1, IRF3, and pIRF3 protein levels. (**j**–**o**) Results of greyscale value analysis of Figure 5i. Data shown are means ± SD from three independent experiments. *** *p* < 0.001, ** *p* < 0.01, * *p* < 0.05.

To investigate the effect of FcγRI on the RIG-I/MDA5 signaling pathway and the expression of type I IFNs, we treated PAMs with anti-FcγRI IgG antibodies for the indicated times. Then, we analyzed the activation status of key molecules in the RIG-I/MDA5 pathway and the levels of IFN-α and IFN-β. As shown in Figure 5, the transcript and protein levels of IFN-α, IFN-β, RIG-I, MDA5, MAVS, TRAF3, TBK1, and IRF3 in the anti-FcγRI IgG-treated cell group were significantly reduced at 12 h and 24 h post-treatment compared to those in the RNI-treated cell group. The supernatants of the cell group treated with anti-FcγRI IgG were negative for both IFN-α and IFN-β proteins. There were no significant differences in the expression of IFN-α, IFN-β, RIG-I, MDA5, MAVS, TRAF3, TBK1, and IRF3 between the RNI-treated cell group and the mock-treated cell group at 12 h and 24 h post-treatment. Furthermore, no pTBK and pIRF3 were detected in the anti-FcγRI IgG-treated cell group, RNI-treated cell group, and the mock-treated cell group at 12 h and 24 h post-treatment (Figure 5i). These data suggested that FcγRI might down-regulate the production of type I IFNs by negatively regulating the RIG-I/MDA5 signaling pathway in PAMs.

### 3.6. FcγRI Down-Regulates Poly (I: C)-Induced Production of Type I IFNs by Negatively Regulating Poly (I: C)-Induced Activation of the RIG-I/MDA5 Signaling Pathway in PAMs

**Figure 6:**
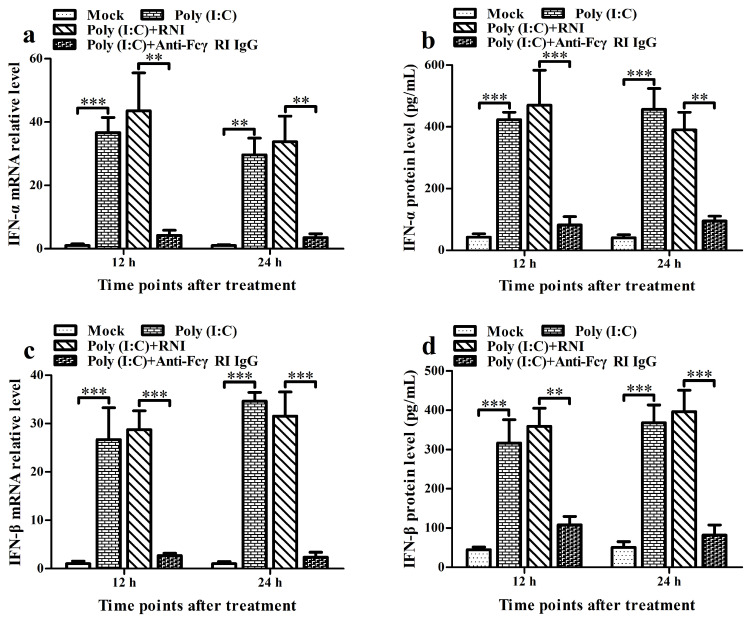

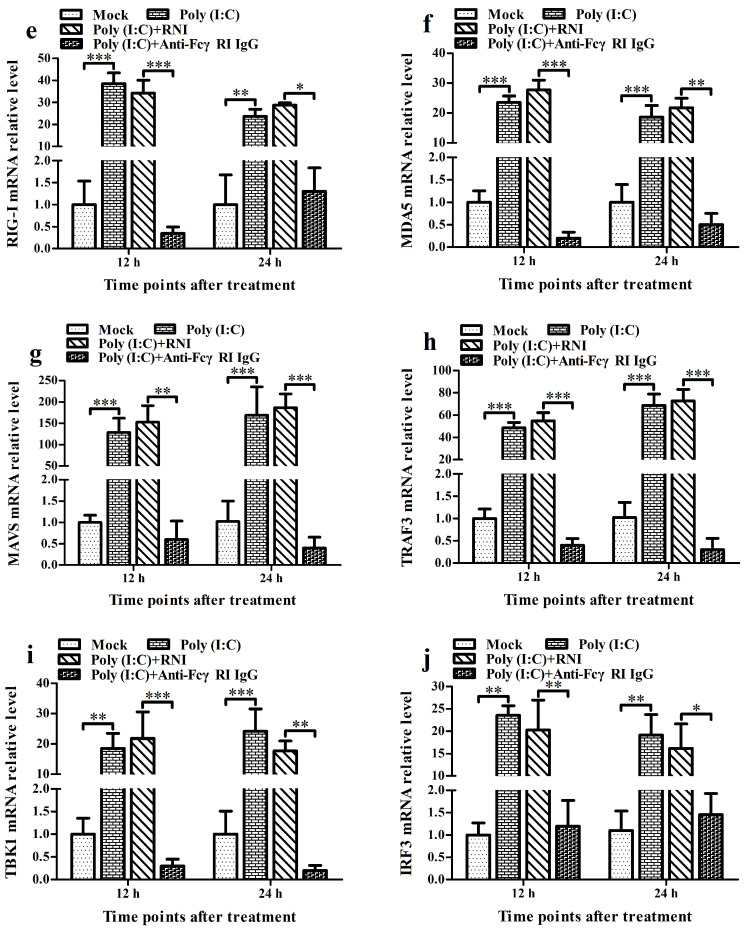

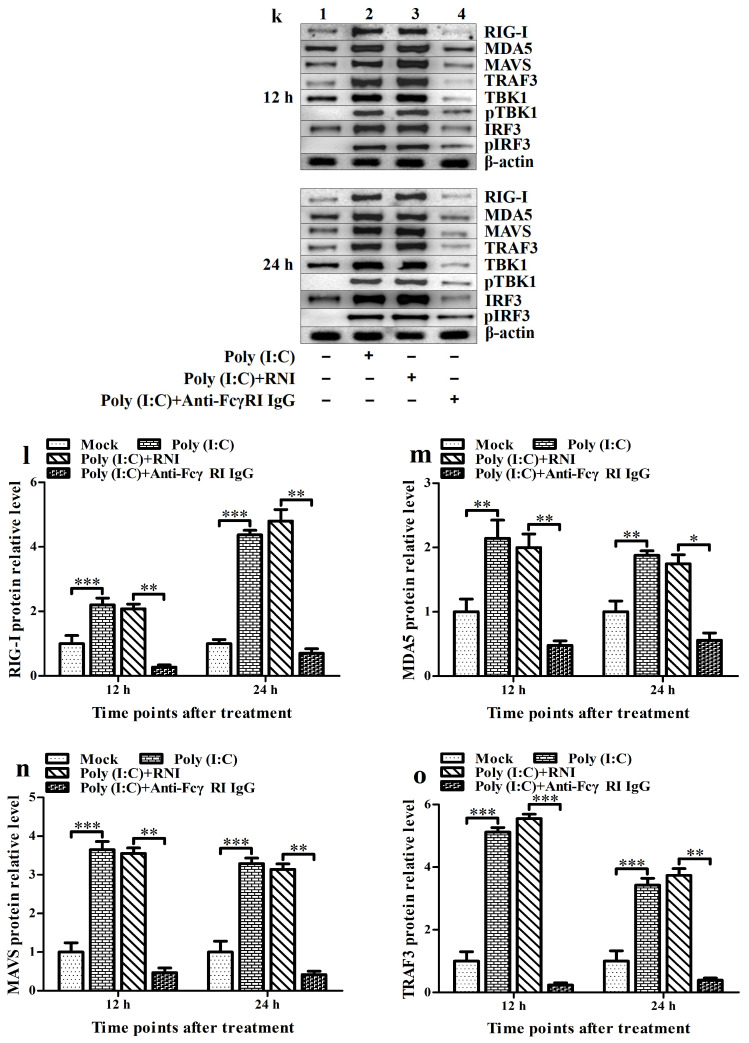

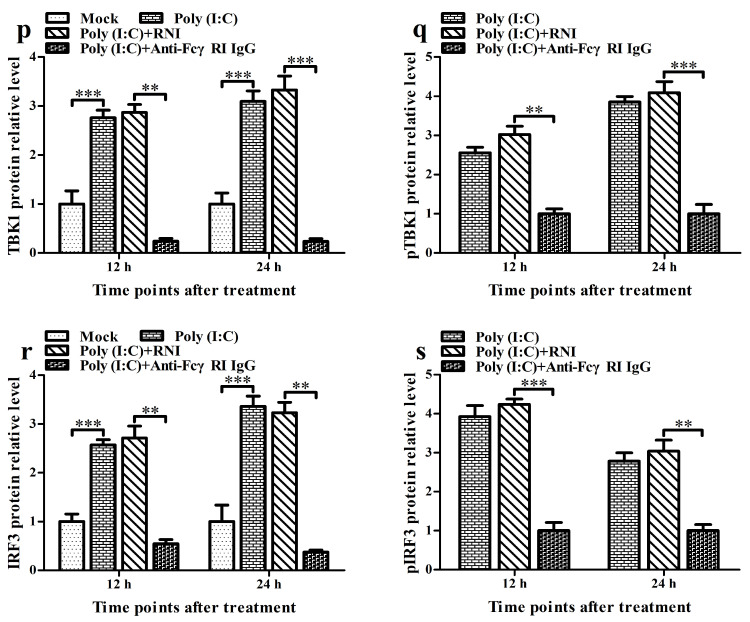
Effect of FcγRI on type I IFN production and RIG-I/MDA5 signaling pathway activation induced by poly (I: C) in PAMs. PAM cells were pre-treated with anti-FcγRI IgG or RNI and then stimulated with poly (I: C). Type I IFN expression levels were detected using relative qRT-PCR and ELISA. Expression and phosphorylation levels of key molecules in the RIG-I/MDA5 signaling pathway were measured by relative qRT-PCR and Western blot. (**a**–**d**) Results of relative qRT-PCR or ELISA analysis of IFN-α and IFN-β production. (**e**–**j**) Results of relative qRT-PCR analysis of RIG-I, MDA5, MAVS, TRAF3, TBK1, and IRF3 mRNA levels. (**k**) Results of Western blot analysis of RIG-I, MDA5, MAVS, TRAF3, TBK1, pTBK1, IRF3, and pIRF3 protein levels. (**l**–**s**) Results of greyscale value analysis of Figure 6k. Data shown are means ± SD from three independent experiments. *** *p* < 0.001, ** *p* < 0.01, * *p* < 0.05.

To investigate the effect of FcγRI on the activation of the RIG-I/MDA5 signaling pathway and the expression of type I IFNs induced by poly (I: C), we used poly (I: C) to stimulate PAMs pre-treated with anti-FcγRI IgG antibody and then analyzed the activation status of RIG-I/MDA5 pathway key molecules as well as the levels of IFN-α and IFN-β. As shown in Figure 6, the transcript and protein levels of IFN-α, IFN-β, RIG-I, MDA5, MAVS, TRAF3, TBK1, and IRF3 in the poly (I: C)-stimulated cell group by anti-FcγRI IgG pre-treatment were significantly decreased at 12 h and 24 h post-stimulation compared to those in the poly (I: C)-stimulated cell group by RNI pre-treatment (Figure 6a–p,r). Simultaneously, the levels of pTBK and pIRF3 in the poly (I: C)-stimulated cell group by anti-FcγRI IgG pre-treatment were significantly less than those in the poly (I: C)-stimulated cell group by RNI pre-treatment at 12 h and 24 h after stimulation (Figure 6k,q,s). However, there were no significant differences in the expression of IFN-α, IFN-β, RIG-I, MDA5, MAVS, TRAF3, TBK1, and IRF3, and the levels of pTBK and pIRF3 between the poly (I: C)-stimulated cell group by RNI pre-treatment and the poly (I: C)-stimulated cell group. These data suggested that FcγRI might down-regulate poly (I: C)-induced production of type I IFNs by negatively regulating poly (I: C)-induced activation of the RIG-I/MDA5 signaling pathway in PAMs.

### 3.7. FcγRI Down-Regulates PRRSV-Induced Production of Type I IFNs by Negatively Regulating PRRSV-Induced Activation of the RIG-I/MDA5 Signaling Pathway in PAMs

**Figure 7:**
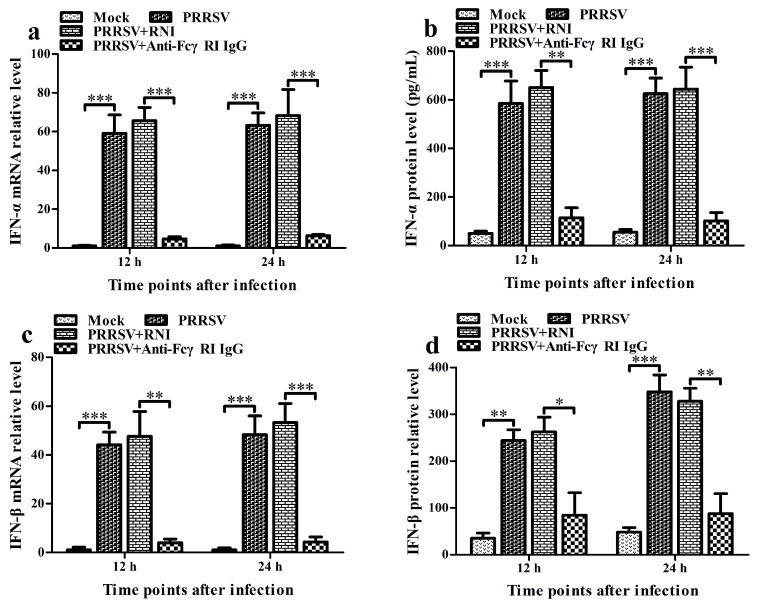

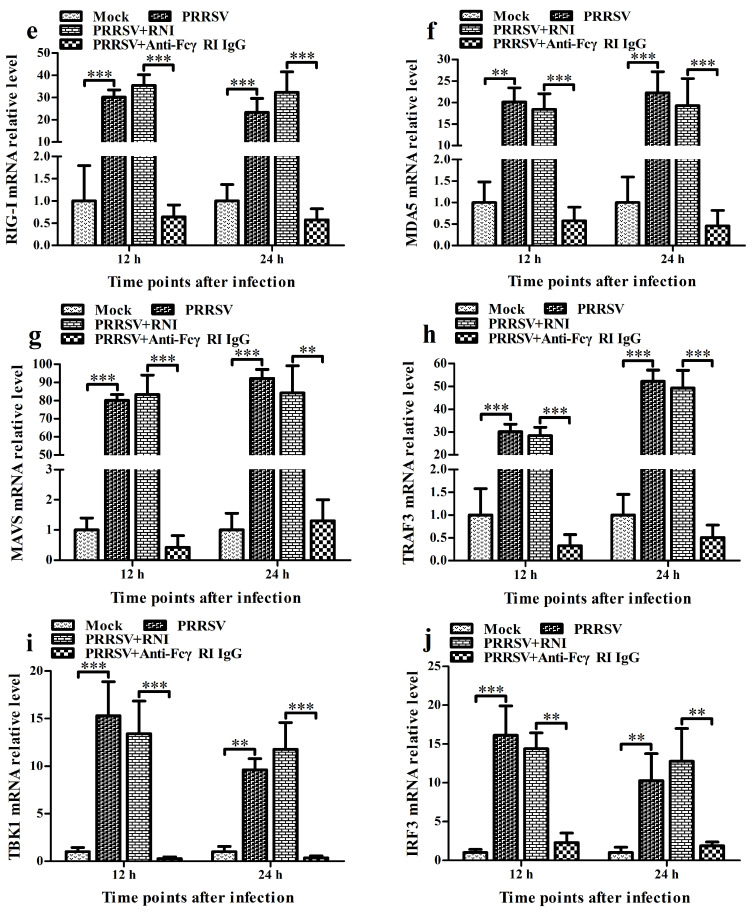

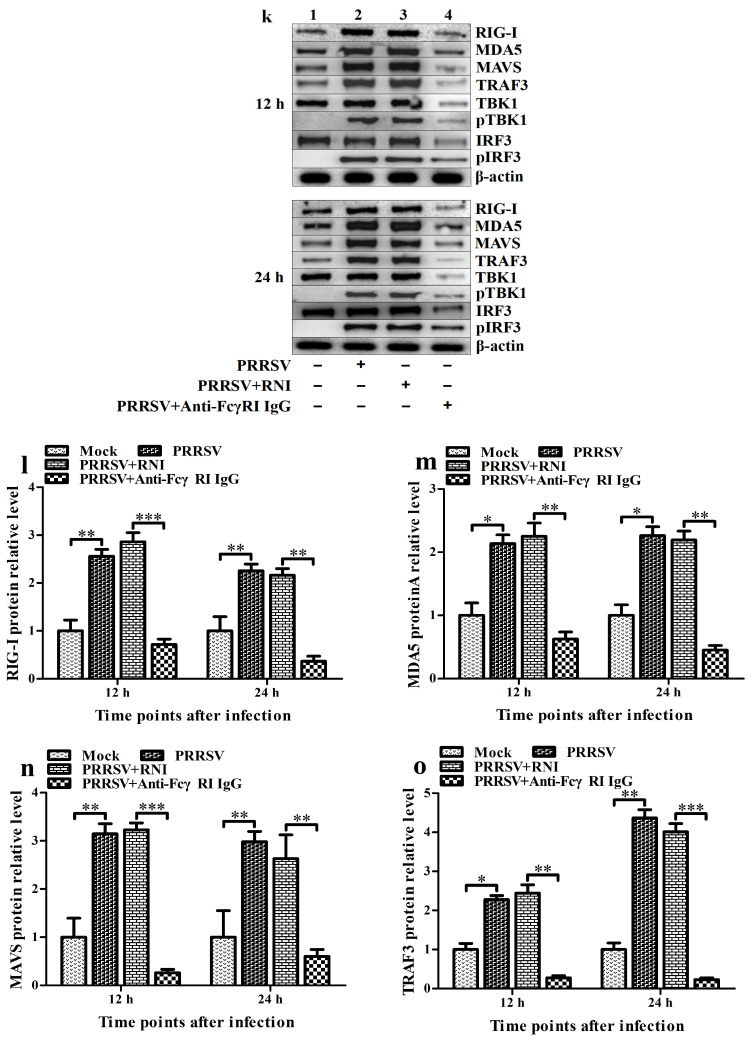

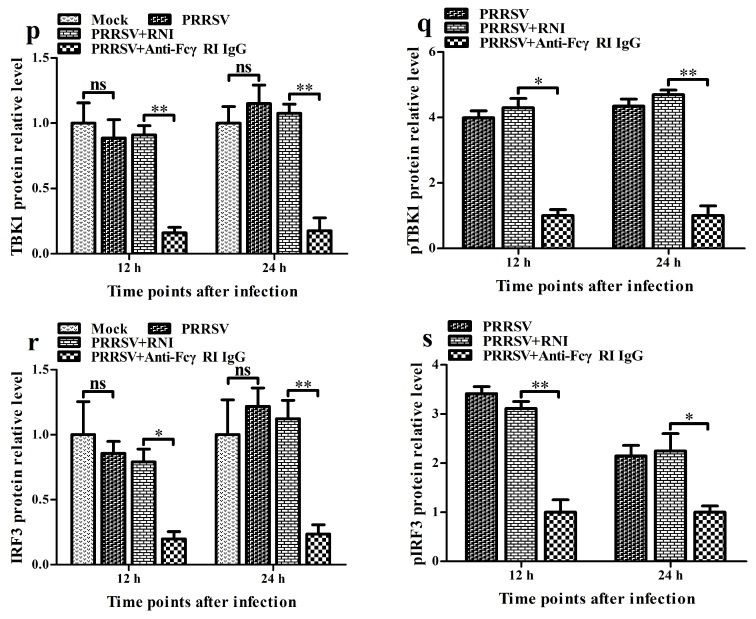
Effect of FcγRI on type I IFN production and the RIG-I/MDA5 signaling pathway activation induced by PRRSV in PAMs. PAM cells were pre-treated with anti-FcγRI IgG or RNI and then infected with PRRSV. Type I IFN expression levels were detected using relative qRT-PCR and ELISA. Expression and phosphorylation levels of key molecules in the RIG-I/MDA5 signaling pathway were measured by relative qRT-PCR and Western blot. (**a**–**d**) Results of relative qRT-PCR or ELISA analysis of IFN-α and IFN-β production. (**e**–**j**) Results of relative qRT-PCR analysis of RIG-I, MDA5, MAVS, TRAF3, TBK1, and IRF3 mRNA levels. (**k**) Results of Western blot analysis of RIG-I, MDA5, MAVS, TRAF3, TBK1, pTBK1, IRF3, and pIRF3 protein levels. (**l**–**s**) Results of greyscale value analysis of Figure 7k. Data shown are means ± SD from three independent experiments. *** *p* < 0.001, ** *p* < 0.01, * *p* < 0.05, “ns” indicating no significant difference.

To investigate the effect of FcγRI on the activation of the RIG-I/MDA5 signaling pathway and the expression of type I IFNs induced by PRRSV, we used PRRSV to infect PAMs pre-treated with anti-FcγRI IgG antibody. Then, we analyzed the activation status of key molecules in the RIG-I/MDA5 pathway and the levels of IFN-α and IFN-β. As shown in Figure 7, the transcript and protein levels of IFN-α, IFN-β, RIG-I, MDA5, MAVS, TRAF3, TBK1, and IRF3 in the PRRSV-infected cell group by anti-FcγRI IgG pre-treatment were significantly declined at 12 h and 24 h post-infection compared to those in the PRRSV-infected cell group by RNI pre-treatment (Figure 7a–p,r). Concurrently, the levels of pTBK and pIRF3 in the PRRSV-infected cell group by anti-FcγRI IgG pre-treatment were significantly lower than those in the PRRSV-infected cell group by RNI pre-treatment at 12 h and 24 h after infection (Figure 7k,q,s). However, there were no significant differences in the expression of IFN-α, IFN-β, RIG-I, MDA5, MAVS, TRAF3, TBK1, and IRF3, and the levels of pTBK and pIRF3 between the PRRSV-infected cell group by RNI pre-treatment and the PRRSV-infected cell group. These data suggested that FcγRI might down-regulate PRRSV-induced production of type I IFNs by negatively regulating PRRSV-induced activation of the RIG-I/MDA5 signaling pathway in PAMs.

### 3.8. FcγRI Promotes the Proliferation of PRRSV in PAMs

**Figure 8:**
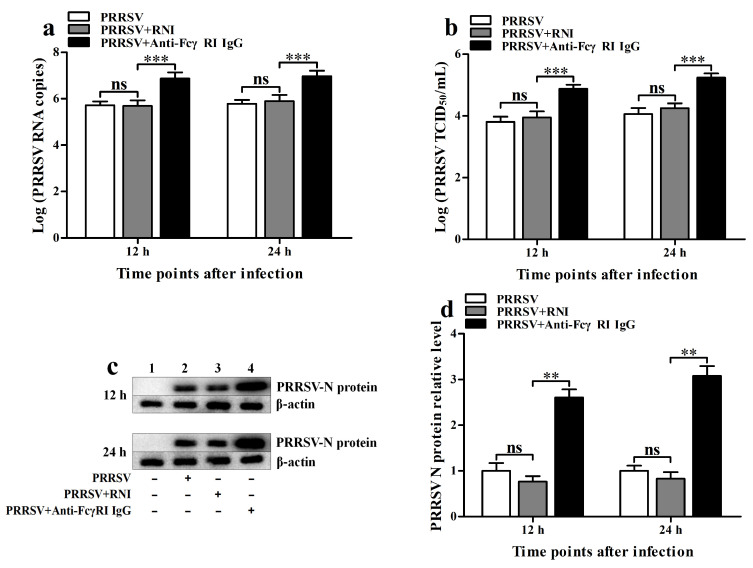
Effect of FcγRI on the proliferation of PRRSV replication in PAMs. PAM cells were pre-treated with anti-FcγRI IgG or RNI and then infected with PRRSV. Real-time RT-PCR, virus titration, and Western blot determined PRRSV yields. (**a**) Results of real-time RT-PCR detection of PRRSV RNA copy numbers. (**b**) Results of virus titration of PRRSV TCID_50_ infection titers. (**c**) Results of Western blot analysis of PRRSV N protein levels. (**d**) Results of greyscale value analysis of Figure 1c. Data shown are means ± SD from three independent experiments. *** *p* < 0.001, ** *p* < 0.01, “ns” indicating no significant difference.

To assess the effect of FcγRI on the replication of PRRSV, we used PRRSV to infect PAMs that had been pre-treated with anti-FcγRI IgG antibody and then detected viral production. As shown in Figure 8, PRRSV RNA copy numbers (Figure 8a) and PRRSV TCID_50_ infection titers (Figure 8b) in PRRSV-infected cells by anti-FcγRI IgG pre-treatment were significantly higher than those in the PRRSV-infected cell group by RNI pre-treatment at 12 h and 24 h post-infection. There were no significant differences in the levels of PRRSV production between the PRRSV-infected cell group by RNI pre-treatment and the PRRSV-infected cell group at 12 h and 24 h after infection. The immunoblot analysis of PRRSV N protein levels in PAMs further supported these results (Figure 8c,d). These data suggested that FcγRI facilitated the replication of PRRSV in PAMs.

## 4. Discussion

Type I IFNs (IFN-α/β) are key antiviral components of the innate immune system. When the PRRs on cells sense the invading pathogens, the innate immune system is activated. RLRs and TLRs are two important host PRRs for RNA viruses. Activation of the RLR or TLR pathway ultimately initiates the expression of type I IFNs [2,40]. As is well known, type I IFNs are usually under strict control during viral infections. PRRSV is a significant economic pathogen affecting the global pig farming industry. A typical immunological characteristic of the virus is its strong inhibitory effect on IFN-α and IFN-β in the infected hosts [41,42]. However, some reports have also indicated that different PRRSV strains may produce distinct patterns of type I IFN release [43,44,45]. Therefore, the infection outcomes and immune responses may vary after infection with different strains. In this study, poly (I: C) was used as a positive control to detect the effect of the PRRSV AF-9 strain on the RLR (RIG-I/MDA5) pathway in PAMs. Enhanced secretion of IFN-α and IFN-β was observed in PAMs infected with AF-9 or stimulated with poly (I: C), which was consistent with previous papers [28,40,45,46,47]. Moreover, AF-9 strain infection or poly (I: C) stimulation led to upregulation of the production of RIG-I, MDA5, MAVS, TRAF3, and TBK1 and phosphorylation of TBK1 and IRF3 in PAMs. These data suggested that PRRSV might induce the secretion of type I IFNs by activating the RIG-I/MDA5 pathway in PAMs. The discovery of the AF-9 strain as an IFN inducer could contribute to the research on PRRSV interfering with the IFN pathway.

ADE is an event in which preexisting poorly neutralizing antibodies against a virus enhance viral infection. When designing active and passive immunization strategies to prevent disease progression, the ADE effect should be carefully considered [16]. However, the detailed cellular mechanisms of ADE are not well-characterized. Early studies showed that RRV-ADE infection reduced the levels of IFN-β by downregulating the transcriptional expression of transcription factors IRF1 and NF-κB in macrophages [48,49]. Subsequent reports indicated that ADE of DENV infection abrogates the production of IFN-β by disrupting the RIG-I/MDA5 or TLR signaling pathway, resulting in suppression of IFN antiviral responses [18,23,50,51]. Here, the inhibition of IFN-α and IFN-β levels was also observed in PRRSV-ADE-infected PAMs relative to those that were PRRSV-infected PAMs, which was similar to previous studies [28,35,36,37]. Furthermore, in contrast to normal PRRSV infection, with ADE in PRRSV infection, the inhibition of RIG-I, MDA5, MAVS, TRAF3, IRF3, and TBK1 gene expression levels and a reduction in phosphorylation levels of TBK1 and IRF3 in PAMs occurred. These data suggest that ADE infection of PRRSV may prevent the release of type I IFNs by blocking the activation of the RIG-I/MDA5 pathway in PAMs. Because type I IFNs can restrict the replication and spread of PRRSV [52]. Therefore, this ADE provides an efficient route for PRRSV to establish an infection cycle in macrophages. Blocking the ADE pathway of type I IFN downregulation could be critical to inhibiting the formation of PRRSV disease.

FcγRs play an essential role in the innate immune system. FcγRs can affect the inflammatory response by controlling the balance between proinflammatory and anti-inflammatory cytokines. For example, the ligation of FcγRI on mouse macrophages in vivo and in vitro can reverse the proinflammatory response by inducing an upregulation of interleukin-10 (IL-10), while mutually inhibiting the production of IL-12 [53]. Similar results have also been demonstrated in another study [54]. In addition, limited evidence suggests that FcγRs can also influence the innate antiviral response by regulating the expression of IFNs. For instance, human FcγRIIa mediates the inhibition of type I and III IFN production induced by RIG-I/MDA5 and TLR3 in various myeloid immune cells [14]. Porcine FcγRI and FcγRIII have also been shown to inhibit the levels of type I IFNs [15,28]. However, the responsible suppression mechanisms are still largely unclear. This study showed that activating FcγRI in PAMs with an anti-FcγRI IgG antibody not only significantly inhibited the synthesis of antiviral cytokines IFN-α and IFN-β but also significantly reduced the production levels of factors related to the RIG-I/MDA5 pathway, including RIG-I, MDA5, MAVS, TRAF3, TBK1, and IRF3. Further, pTBK and pIRF3 were not detected in FcγRI-activated PAMs. Moreover, the activation of FcγRI in PAMs downregulated the secretion of IFN-α and IFN-β induced by poly (I: C) through suppressing the upregulation of RIG-I, MDA5, MAVS, TRAF3, TBK1, and IRF3 production, as well as the phosphorylation of TBK and IRF3 induced by poly (I: C). These data suggested that FcγRI might counteract the synthesis and release of type I IFNs by inhibiting the RIG-I/MDA5 pathway in PAMs. These results will contribute to deepening our understanding of the role of the FcγRI signaling in the inhibition of innate antiviral immunity.

FcγRs are required for ADE infection of many viruses. Activating FcγRs promote ADE during viral infection, while the inhibitory FcγRIIb plays a negative regulatory role in this process [16,55]. Additionally, Ubol et al. have pointed out that the engagement of FcγRIIa is not only a critical part for the entry of infectious DENV-antibody complexes into THP-1 monocytes, but also plays a crucial role in the process of antiviral immune evasion by turning off the production of type I IFNs [23]. To identify the role of FcγRI in the inhibition of type I IFN expression by PRRSV-ADE infection, we silenced FcγRI in PAMs using siRNA against FcγRI. Then we infected the cells with enhanced virus-antibody complexes (PRRSV-ICs). As previously demonstrated by our team and Shi et al. [28,36,56], knockdown of FcγRI effectively eliminates the ADE phenomenon of PRRSV in PAMs. These findings suggested that FcγRI was responsible for ADE infection of PRRSV. Concurrently, we also observed that the activation of FcγRI in PAMs reduced the production of IFN-α and IFN-β induced by PRRSV by counteracting the upregulation of RIG-I, MDA5, MAVS, TRAF3, and TBK1 expression caused by PRRSV and the increase in the phosphorylation levels of TBK and IRF3 induced by PRRSV, and enhanced the growth of PRRSV in activated PAMs. These data suggested that FcγRI might inhibit the release of type I IFNs induced by PRRSV by negatively regulating the activation of the RIG-I/MDA5 pathway caused by PRRSV, thereby promoting the proliferation of PRRSV in PAMs. Taken together, these results indicated that PRRSV infection of PAMs via FcγRI (ADE pathway) might antagonize the secretion of type I IFNs through interfering with the intracellular RIG-I/MDA5 signaling pathway, shifting it from an antiviral mode to a virus-promoting mode, thereby resulting in enhanced PRRSV infection. In conclusion, this study revealed that ADE infection of PRRSV evaded the antiviral state within host cells through an immunosuppressive loop mediated by FcγRI, which provided new insights into mechanisms of ADE and offered a potential new cellular target for the development of therapeutic strategies for ADE-related diseases.

## 5. Conclusions

The present study showed that PRRSV infection alone induced the secretion of type I IFNs in PAMs by activating the RIG-I/MDA5 pathway. In contrast, PRRSV infection via the FcγRI-mediated ADE pathway inhibited the production levels of type I IFNs in PAMs by blocking the activation of the RIG-I/MDA5 signaling pathway, thereby facilitating the growth of PRRSV. This study revealed the potential mechanism by which PRRSV evades the innate antiviral response through the FcγRI-mediated ADE infection pathway.

## Figures and Tables

**Table 1:** Relative qRT-PCR primers.

Name ^#^	Sequence (5′–3′)
IFN-α F IFN-α R	GGATCAGCAGCTCAGGG GAGGGTGAGTCTGTGGAAGTA
IFN-β F IFN-β R	CAACAAAGGAGCAGCAAT TGGAGCATCTCGTGGATA
RIG-I F RIG-I R	AGCACCTCATACTTACAGCCC CCTTCCCCTTTCGTCCTTGT
MDA5 F MDA5 R	TGACGAATGCCATCACACCA TTGGCTTGCTTTTTGGCTCC
MAVS F MAVS R	AGTGTCCCGTGTCTACCAGA GGACAGGCATGGGGTAACTT
TRAF3 F TRAF3 R	GCCAGGTCCCAATGATCACA GGGGCATTGACACACTCTGA
TBK1 F TBK1 R	TTTAGATGGGGATGCCAGCG CCATCGTATCCCCTTTCGCA
IRF3 F IRF3 R	ATCGAAGGAAGCAGACGCTC AACCTTGACCATCACCAGCC
FcγRI F FcγRI R	TGAAACAAAGTTGCTCCCA GCTGCGCTTGATGACCT
β-actin F β-actin R	CGGGACATCAAGGAGAAGC CTCGTTGCCGATGGTGATG

^#^ F, forward primer; R, reverse primer.

## Data Availability

The available data are found in the paper.
